# Three dimensional metal/N-doped nanoplate carbon catalysts for oxygen reduction, the reason for using a layered nanoreactor

**DOI:** 10.1038/s41598-018-21782-3

**Published:** 2018-02-21

**Authors:** Mohammad Yeganeh Ghotbi, Arash Javanmard, Hassan Soleimani

**Affiliations:** 1 Materials Engineering Department, Faculty of Engineering, Malayer University, Malayer, Iran; 2 Fundamental and Applied Science Department, Universiti Teknologi PETRONAS, 31750 Seri Iskandar, Malaysia

## Abstract

A layered nanoreactor (zinc hydroxide gallate/nitrate nanohybrid) has been designed as a nano-vessel to confine the gallate/nitrate reaction inside zinc hydroxide layers for production of metal/nitrogen-doped carbon catalysts. Metals (Fe^2+^, Co^2+^ and Ni^2+^) doped and bare zinc hydroxide nitrates (ZHN) were synthesized as the *α*-phase hydroxide hosts. By an incomplete ion-exchange process, nitrate anions between the layers of the hosts were then partially replaced by the gallate anions to produce the layered nanoreactors. Under heat-treatment, the reaction between the remaining un-exchanged nitrate anions and the organic moiety inside the basal spacing of each nanohybrid plate resulted in obtaining highly porous 3D metal/nitrogen-doped carbon nanosheets. These catalysts were then used as extremely efficient electrocatalysts for catalyzing oxygen reduction reaction (ORR). This study is intended to show the way to get maximum electrocatalytic activity of the metal/N-doped carbon catalysts toward the ORR. This exceptionally high ORR performance originates from the increased available surface, the best pore size range and the uniform distribution of the active sites in the produced catalysts, all provided by the use of new idea of the layered nanoreactor.

## Introduction

When non-precious metal catalysts (NPMCs) were introduced, a promising future of applying them for catalyzing the slow oxygen reduction reaction (ORR) of polymer electrolyte fuel cell (PEFC) cathodes and the commercial use of the cost-effective devices based on PEFCs was created^1–5^. The development of highly efficient NPMCs could result in solving the issues faced with the use of the noble metals of Pt and Pd as the benchmark electrocatalysts for the ORR. These issues are addressed as high costs and scarcity of the noble metals in addition to cathode catalyst oxidation, catalyst migration and loss of surface area of electrocatalysts, leading to overpotentials and durability harms^6–8^.

One of the most important NPMCs is the nitrogen-doped carbon material (NCM) since nitrogen within carbon structure acts as an n-dopant and donates electrons to the carbon and facilitates the ORR^2,9,10^. In accompanied with the nitrogen, when some transition metals in particular Ni, Co, Fe and also Cu is added within carbon structure, they act as active electrocatalyst centers via formation of the structures as metal-N_4_ and they consequently enhance the ORR activity, improve performance and durability of the cell as front-runners^1,2,5,11–15^.

In recent years, several different types of organic and inorganic nanoreactors were introduced. Nanoreactors are nano size matrices, confining discrete chemical reactions from the surrounding bulk media^16–18^. These nanosized containers are either synthetically produced (for instance, nano porous, tubular and hollow nanomaterials with various geometries and diameters) or they are inherently composed of nano pores and channels (for instance, proteins, lipids and zeolites, etc)^16–20^. This confinement offers the control of a chemical reaction to make a particular product such as a new class of nanomaterials and/or gains a new insight into fundamental basics of physics and chemistry of the material^16,19^.

In our previous works, we showed that the NCMs could be synthesized by designing the organic-inorganic layered nanoreactors^21,22^. After heat-treatment process of the nanoreactor, an organic-nitrate reaction occurred inside the layers, resulting in the production of the NCMs. Due to the fact that other authors have used the organic compounds containing nitrogen for production of the NCMs^1,2,23–25^, the nanoreactor idea could help us to use every organic anions which can only be intercalated between the inorganic layers. Herein, we want to show how a layered nanoreactor can be used for preparing the metal/N-doped carbon catalysts. The reason why we use a layered nanoreactor is because we want to demonstrate that the method is the preferred approach. We show in details, how three-dimensional metal/N-doped nanoplate carbon catalysts with excellent dispersity of the nitrogen and metal dopants within carbon structure, high surface area and appropriate porosity, and, specifically high-electrocatalytic performance for the ORR can be obtained by the idea of the layered nanoreactor.

## Results

**Figure 1:**
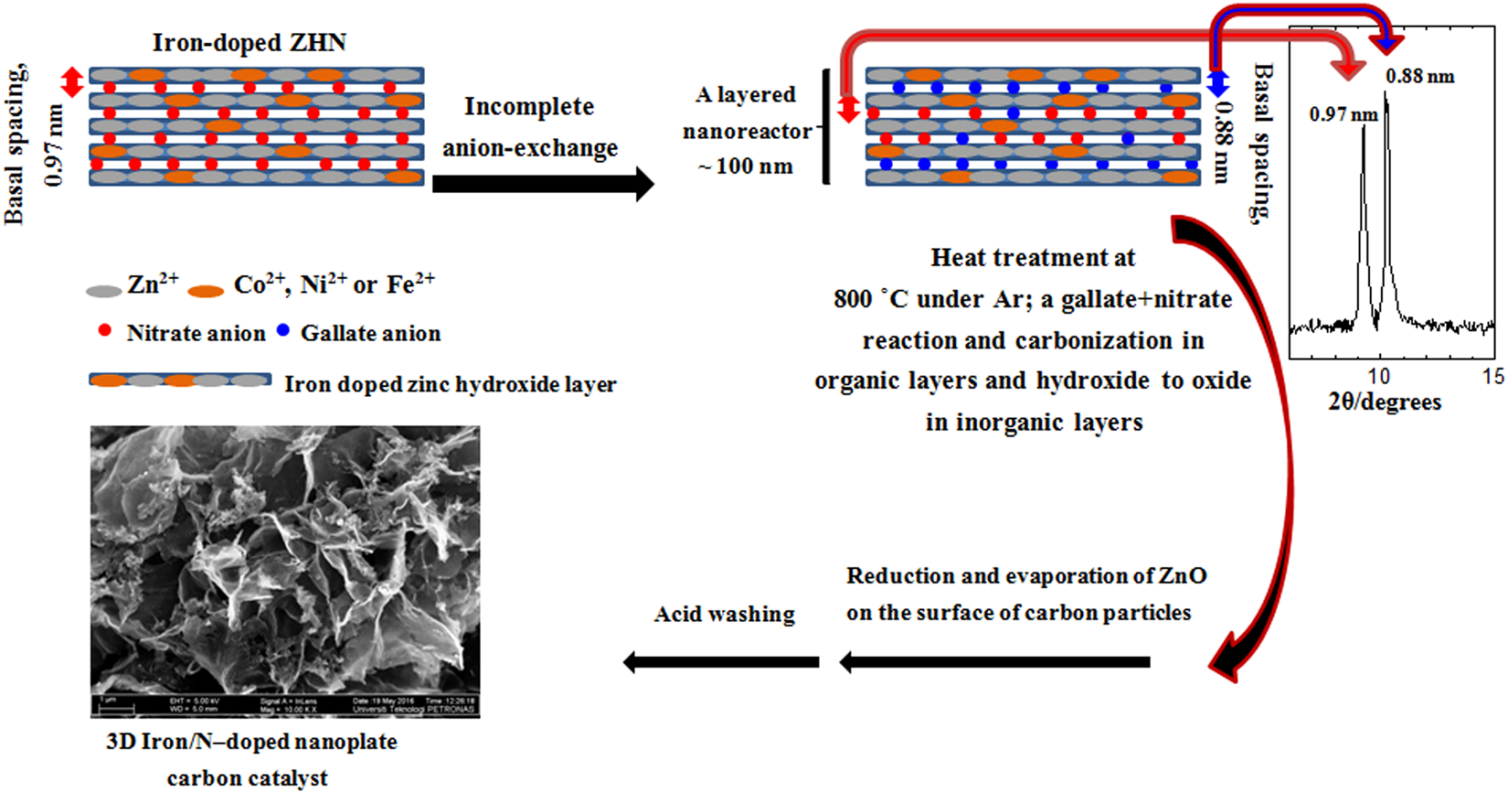
Schematic diagram of the synthesis of Fe/N-doped carbon catalyst.

The catalyst synthesis as a simple schematic diagram is shown in Fig. 1. We started with an *α*-phase material, namely zinc hydroxide nitrate (Zn_5_(OH)_8_(NO_3_)_2_.2H_2_O) (Card no, 24–1460) as a layered host. This is a layered material with positively charged brucite-type layers constructed solely with one type of cation (Zn^2+^). The positive charges within the brucite-type layers are neutralized by nitrate anions positioned between the layers^26,27^. Each layer can be doped by partial replacement of Zn^2+^ for other divalent M^2+^/trivalent M^3+^ cations^27^. In this work, undoped zinc hydroxide nitrate (ZHN) and doped zinc hydroxide nitrates with 2% (molar) Fe^2+^, Co^2+^ and Ni^2+^ were synthesized. The ZHNs were chosen as the inorganic layered hosts and gallate anion as an organic guest, which was encapsulated into the intergallery of the hosts to produce the organic-inorganic layered nanohybrids using simple ion-exchange method^27^. Herein (see Fig. 1), partial anion-exchanging was done due to the co-existence of both nitrate (as a nitrogen source) and gallate (as a carbon source) anions between the inorganic layers. Heat-treating the resultant nanohybrids at 800 °C could lead to prepare N-doped carbon catalysts^21,22^. Moreover, the doping of the initial layered hosts with some transition metals was for incorporating the metallic dopants within upcoming N-doped carbon structures and, therefore, obtaining the metal/N-doped carbon catalysts. Finally, excess metal/metal oxide should be removed by acid etching of the produced carbon catalysts in an HCl solution. In fact, the undissolved metal dopants are encapsulated inside the carbon layers^9,12,28,29^.

Firstly, we want to show how 3D metal/N-doped nanoplate carbon catalysts are produced by using an organic-inorganic layered nanoreactor. Due to the best performing catalyst was the carbon derived from FZHNG nanohybrid (FCNG, Fe/N-doped carbon catalyst), the physicochemical results about formation of the carbon catalysts were oriented toward FCNG.

**Figure 2:**
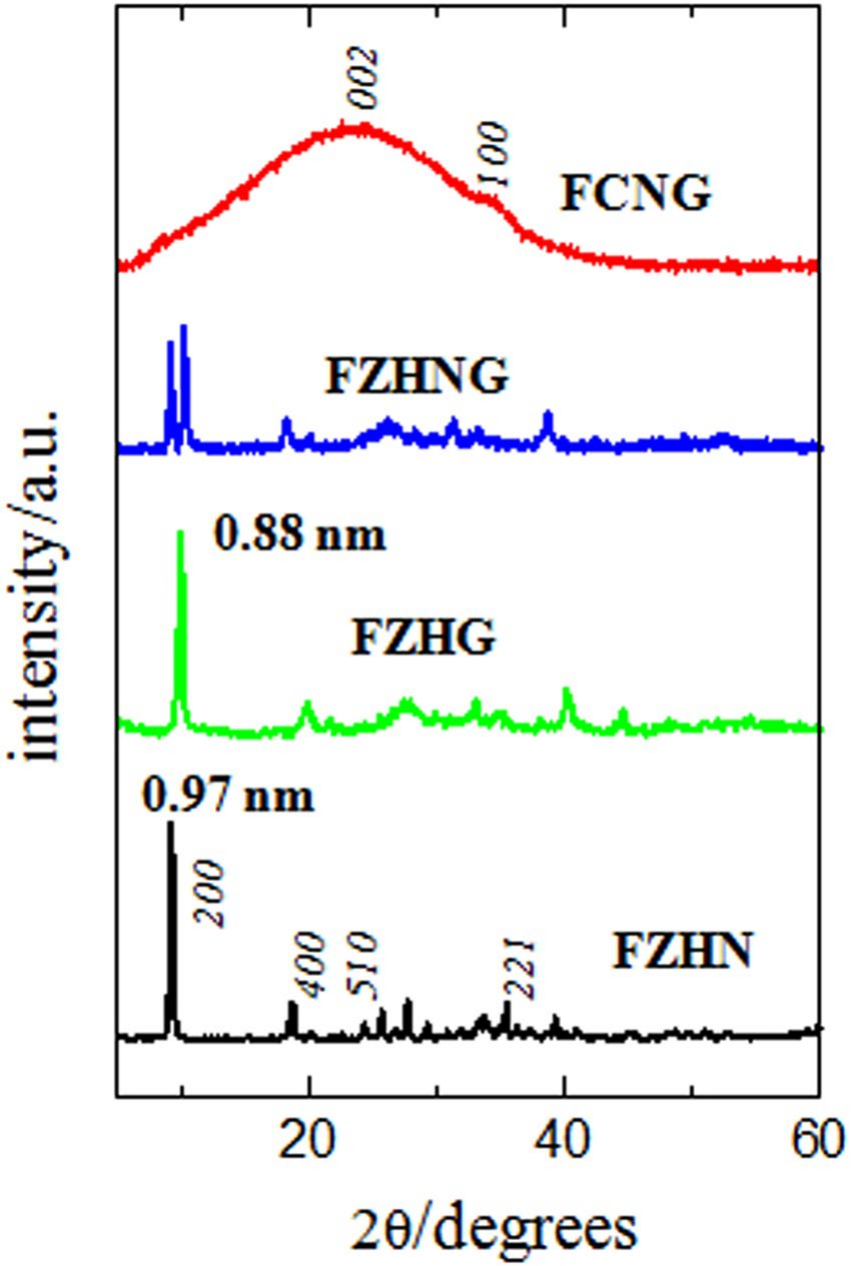
XRD patterns for the as-synthesized FZHN, its nanohybrid (completely ion-exchanged) with gallate anion (FZHG), its nanohybrid (incompletely ion-exchanged) with gallate anion (FZHNG) and its resultant carbon catalyst (FCNG).

XRD patterns for the FZHN and also its nanohybrids with gallate anion, completely ion-exchanged (FZHG), incompletely ion-exchanged (FZHNG) and FCNG carbon catalyst are shown in Fig. 2. As observed, three samples of the FZHN, FZHG and FZHNG show the *α*-phase brucite-like structures with the basal spacing peak around 0.97 nm for nitrate and 0.88 nm for gallate anions^22,27^. It should be noted that all peak positions of the undoped and metal-doped ZHN samples were the same in their XRD patterns due to small amounts of the metal doping agents^27^. As seen, the organic anions could be intercalated into the interlayer of the parent material via the anion-exchange process, where gallate anions lie flat with the phenolic hydrogen perpendicular to the ring between the layers^27,30^. The basal spacing peak at 0.97 nm attributed to the parent material (FZHN) can also be observed in the XRD pattern of the FZHNG nanohybrid owing to the incompleteness of the anion-exchange process. It indicates that both anions of gallate and nitrate are simultaneously present inside the interlayer spaces of the FZHNG layers for each plate as illustrated in Fig. 1. Figure S5 shows FTIR spectra for the FZHN, gallic acid, the FZHG and the FZHNG nanohybrids. The FTIR spectrum of the FZHG nanohybrid is composed of spectral band features of both gallic acid and zinc layered hydroxide, but is free of nitrate anion bands^22,27^. For the FZHNG sample, the FTIR spectrum is composed of spectral band features of gallate anions and zinc layered hydroxide in accompanied to nitrate anions due to the presence of the intense peak at 1378 cm^−1^ attributed to υ_3_ of the co-intercalated nitrate anion^31^. It means that both nitrate and gallate anions are concurrently between the inorganic layers for each nanohybrid plate.

As stated earlier, the metal/N-doped carbon catalysts were produced using the heat-treatment process of the nanohybrids via a chemical reaction between nitrate and gallate anions both encapsulated between the inorganic layers. In order to monitor the gallate-nitrate reaction (as illustrated in Fig. S1), thermal and phase analyses were carried out on the FZHN precursor and its FZHNG nanohybrid (see Supplementary information).

Figure 2 also reflects XRD pattern of the obtained FCNG carbon catalyst. A very broad peak around 23° is observed in the pattern, confirming that the catalyst is essentially in amorphous form with disordered graphite structure within the *c*-planes. Figure S5b shows the FTIR spectrum for the FCNG catalyst obtained by heat-treating the FZHNG nanohybrid followed by acid washing. There are two broad bands around 1330 and 1620 cm^−1^ owning to C=N/C–C and C=C/C=O stretching vibrations, respectively^9,14,23^.

**Figure 3:**
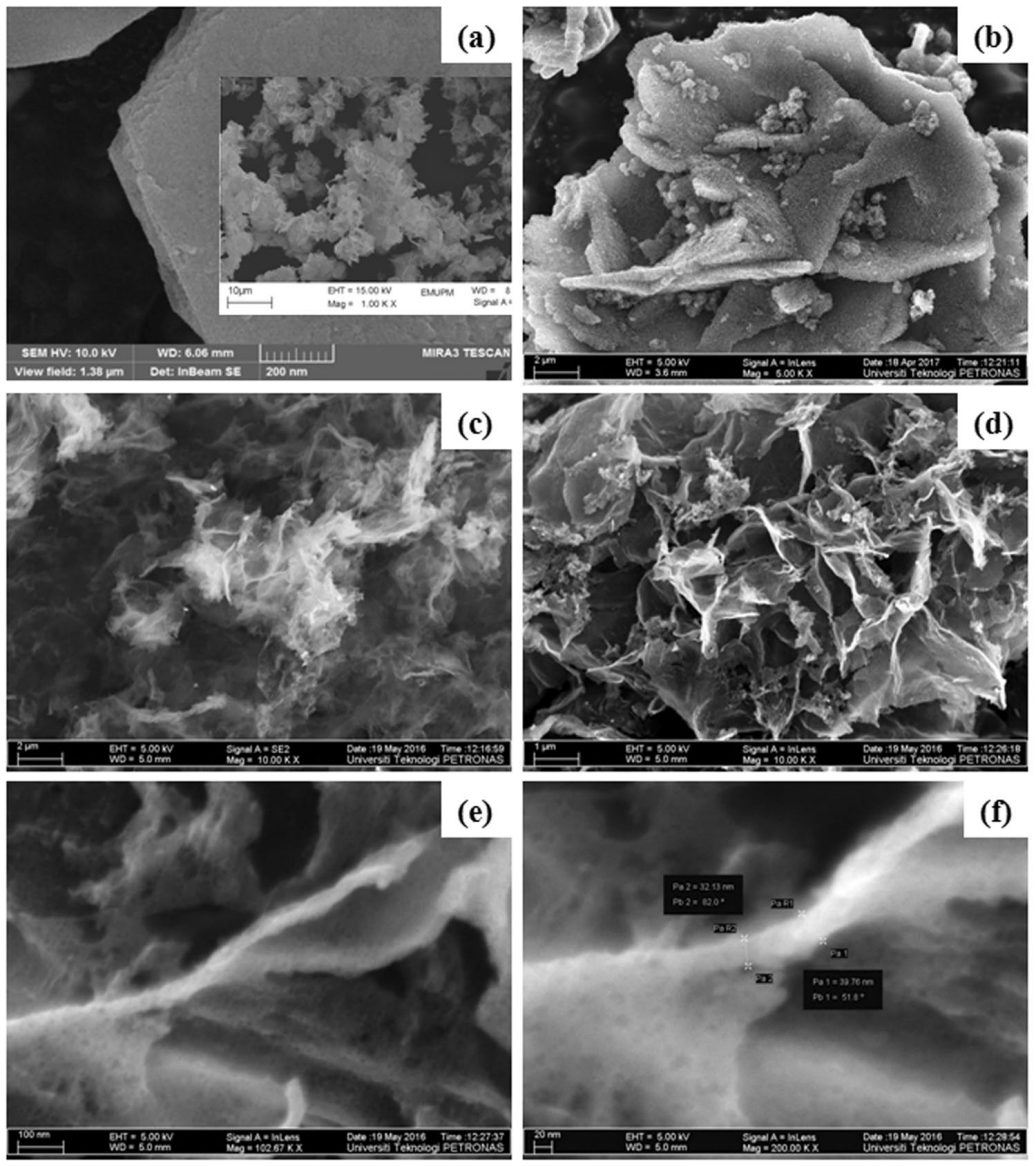
FESEM images of a FZHN plate (inset shows typical surface morphology of a ZHN sample) (**a**), the CG carbon derived from FZHG nanohybrid (**b**) and the FCNG carbon catalyst at different magnifications (**c**–**f**).

The SEM micrograph in Fig. 3a shows the morphology of a nanoplate of the FZHN precursor material, and the micrograph in Fig. 3b shows the carbon material obtained from the heat-treatment of the FZHG nanohybrid (with only gallate anions) followed by acid washing. As obviously seen, the thicknesses of the obtained carbon plates are almost the same with that of the initial precursor plate, *ca*. 100 nm. That is, the produced carbon materials are replicas of the initial plates when the anions between the zinc hydroxide layers are only organic. However, when the anions between the ZHNs are a combination of gallate and nitrate anions, one cannot see the carbon plates with the thicknesses similar to those of the initial plates of the FZHN (Fig. 3c). It seems that each plate is horizontaly split after acid washing (see Fig. S1). As shown in Fig. 3d–f, the thickness of the plates of the FCNG carbon catalyst are less that 40 nm. In addition, the Fig. 3d confirm the three dimensional structure as well as highly porous nature of the FCNG carbon catalyst.

**Figure 4:**
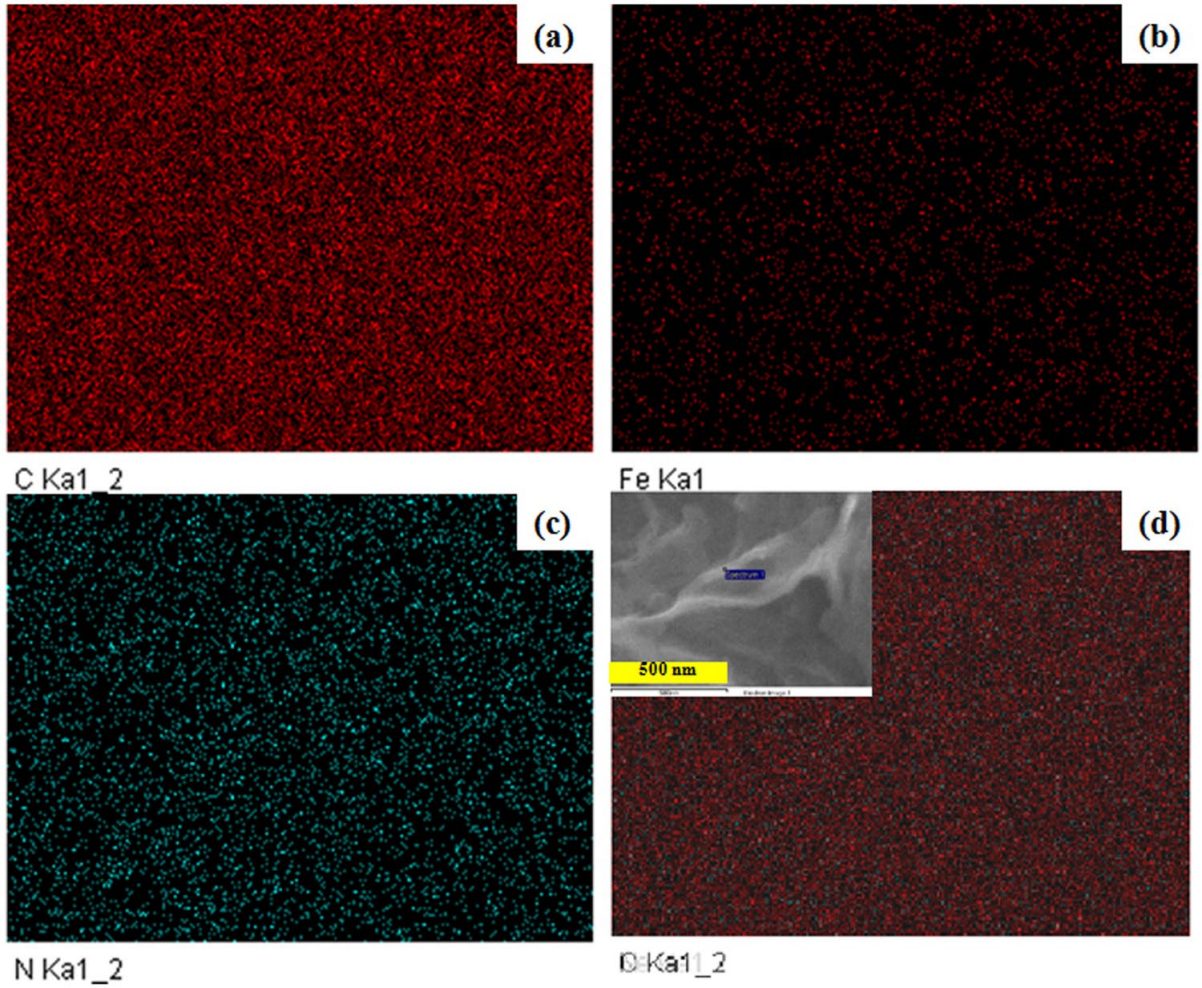
The elemental mapping images of (**a**) carbon, (**b**) iron (**c**), nitrogen and the overlay of the elemental mapping images (**d**).

Elemental mapping of the FCNG was performed to confirm the presence of the nitrogen and iron dopants in the carbon sheets and to evaluate the homogeneous dispersion of the dopant elements. For this end, we selected a very small area within a thin carbon blade (see inset in Fig. 4d) in the sample. A highly uniform distribution of both nitrogen and iron dopants within carbon structure can be seen in this small area (Fig. 4b and c). Moreover, it is well recognized that the nitrogen content is higher than the iron content in the catalyst. Figure 4d shows an overlay image of the C, N and Fe images, and it confirms the uniform dispersion of the dopant elements, again.

If we accept the splitting process of the plates after heat-treatment and acid etching, then the surface area value for the N-doped carbon materials should be higher than that of the undoped carbon material (CG). Figure S7 shows the adsorption-desorption isotherms for the carbon materials of the CG (undoped carbon), and the FCNG (Fe/N-doped carbon) derived from the ZHG and the FZHNG nanohybrids, respectively. These isotherms are of Type IV, and Type H3 hysteresis loops according to IUPAC nomenclature^32^ in which adsorption increases slowly at low relative pressure, and rapidly near the saturated pressure. This is the characteristic of plate-like materials with slit-shaped pores for the carbon samples^32^. The Brunauer-Emmett-Teller (BET) surface area values, mean pore diameters and total pore volumes for the carbon catalysts are shown in Table S1. As seen, the surface area and the pore volume values are respectively, 458 m^2^ · g^−1^ and 1.26 cm^3^ · g^−1^ for the CG and 710 m^2^ · g^−1^ and 2.23 cm^3^ · g^−1^ for the FCNG. That is, the doping carbon material with nitrogen and iron could result in an increase in both surface area and pore volume values for more than 1.5 times. However, the question that remains is: how this happens? According to SEM images, the answer is horizontal splitting each nanohybrid plate after the heat-treatment and the acid leaching processes; the thinner plates have higher surface area values. Barret-Joyner-Halenda (BJH) pore size distribution analysis (Fig. S8) shows a single pore distribution with 2.6 nm pore diameter peak and the MP-method shows mean pore diameters of 2.0 nm for both samples. Due to the fact that the BJH pore size distributions of both samples are almost free of micropores as well as macropores, it is deduced that the pores are dominantly within meso region around micro/meso boundary.

**Figure 5:**
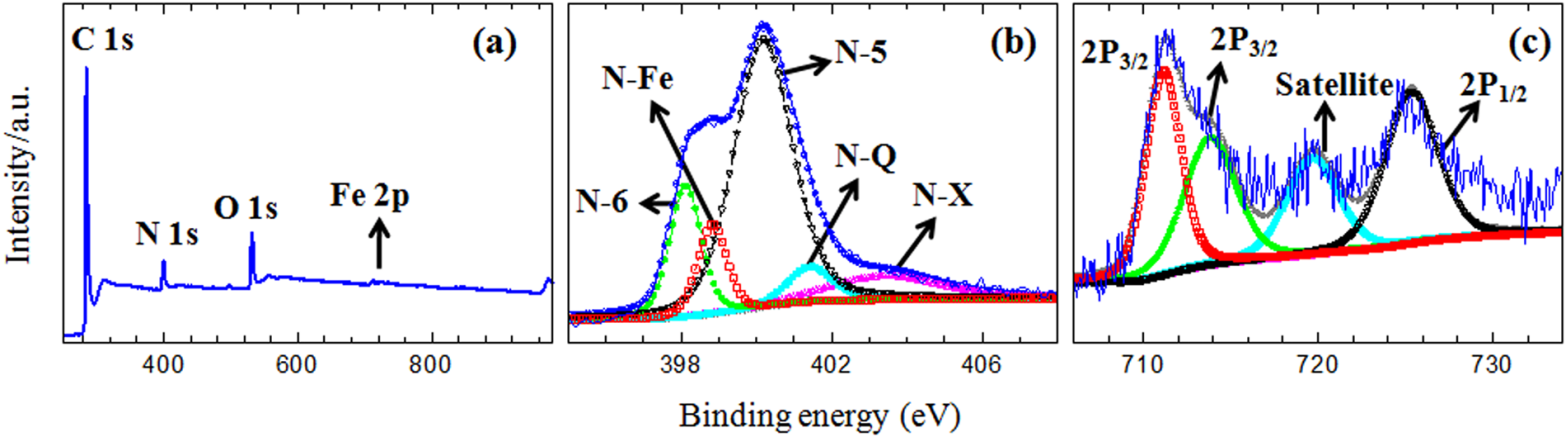
XPS survey scan of the FCNG carbon catalyst (**a**). High resolution N 1 s (**b**) and Fe 2p XPS spectra of the FCNG catalyst (**c**).

X-ray photoelectron spectroscopy (XPS) was carried out to evaluate the surface chemical composition of the FCNG catalyst. Figure 5 shows XPS survey scan of the catalyst. The spectrum contains three sharp photoelectron peaks of C, N and O at binding energies of 284.48 eV (C 1 s), 399.64 eV (N 1 s) and 531.63 eV (O 1 s), and, also a weak peak for Fe located at about 711.84 eV (Fe 2p)^4,12,14,23^. The atomic concentrations of C, N, O and Fe are 83.81, 6.81, 8.97 and 0.37%, respectively. Further analyses were performed on the N 1S and the Fe 2p signals as shown in Fig. 5b and c. The fitting of the N 1 s spectra were resolved into five peaks at 398.12, 398.82, 400.18, 401.43, and 403.4 eV. The peak deconvolution analysis of N 1 s and Fe 2p and their composition ratios are listed in Table S2. It is known that the peaks with the lowest binding energies are due to pyridinic (N-6) nitrogen (398.12 and 398.82 eV), where one N atom with two C atoms are hybridized as *sp*^2^ hybridization^10,33,34^. Pyrrolic nitrogen (N-5) has a higher binding energy peak (400.18 eV) than that of pyridinic, but with the same hybridization, where N is bonded with two carbon and one hydrogen in a pentagonal heterocyclic ring of C atoms. When nitrogen is *sp*^3^ hybridized via three single bonds with carbon atoms in a graphene layer, the peak is observed at 401.73 eV due to quaternary nitrogen (N-Q). Finally, the peak at 403.4 eV is assigned to pyridine oxide or the N^+^-O^−^ (N-X) group composed of one N atom bonded to one oxygen and two carbon atoms. The FCNG catalyst has a high content of the N-5 and N-6 configurations, a low content of the N-X and a very small amount of the N-Q.

The high resolution XPS spectrum of the Fe signal can also be deconvoluted into four peaks at 711.16, 713.85, 719.72 and 725.36 eV. The peaks at 711.16 and 713.85 eV can be attributed to 2P_3/2_ Fe(III) and Fe(II) ions, respectively and the peak at 725.36 eV is ascribed to 2P_1/2_ for both Fe(III) and Fe(II) ions. Moreover, the peak at 719.72 eV is a satellite peak for the above three peaks, an indicating the co-existence of Fe(II) and Fe(III) ions in the sample^12,14,35,36^. The lack of the peak at about 707 eV confirms that the catalyst does not include iron in metallic form^12,29^. The crystalline form of iron cannot be precisely determined, because Fe(II) and Fe(III) contents are almost the same (Table S2) and the FCNG carbon catalyst does not show any XRD peaks due to iron compounds (Fig. 2). However, it was recommended that the peak at 398.82 eV may be corresponded to bonded Fe and N atoms as in-plane Fe–N_4_ centers surrounded by the carbon layers^11,12,33,37^. On the other hand, comparing the XPS and EDS results (Table 1), one can deduce that the iron content on the surface is less than that of the bulk material. It may be due to the acid leaching process, during which acid can penetrate easier into the surface layers as compared to in the carbon bulk. The O 1 s energy peak at 531.63 eV reveals the presence of C=O group on the surface of the carbon catalyst in good agreement with its FTIR spectrum (See Fig. S5b)^10,23^. In fact, the presence of oxygen in the catalyst is due to the oxidized pyridinic N-X nitrogen (9.7%), C=O group on the carbon surface and may be in part due to iron oxide. Of course, since the total amount of oxygen attributed to both N-X and iron oxide is less than 1 percent, the remaining oxygen (≈8%) is due to C=O group on the carbon surface. The energy peak for the C 1 s XPS signal is at 284.48 eV, indicating that the dominant contribution is due to the C sp^2^-hybridized state in graphite structure in agreement with its XRD pattern^23^.

**Table 1:** Obtained data from ORR kinetic and elemental analysis of the carbon catalysts.

Catalyst	ORR onset potential (V)	Tafel slope (mV/dec)	*i*_0_ (A/cm^2^)	D^1/2^*c* (mol/cm^2^ · s^1/2^)	C^d^ (%)	N^d^ (%)	O (%)
CNG	0.86	66.4	4.82 × 10^−5^	8.02 × 10^−8^	84.19	5.83	9.93
NCNG^a^	0.94	58.3	7.62 × 10^−5^	8.46 × 10^−8^	82.63	5.64	10.22
CCNG^b^	0.97	48.6	1.02 × 10^−4^	9.32 × 10^−8^	82.93	5.47	10.11
FCNG^c^	1.00	43.2	1.52 × 10^−4^	1.19 × 10^−7^	82.02	5.72	10.63
Pt/C	0.98	45.4	4.61 × 10^−4^	6.74 × 10^−6^	90	—	—

^a^NCNG consists of 1.51 wt% nickel.

^b^CCNG consists of 1.44 wt% cobalt.

^c^FCNG consists of 1.57 wt% iron.

^d^The measurements were done by CHNS instrument (Leco, USA).

**Figure 6:**
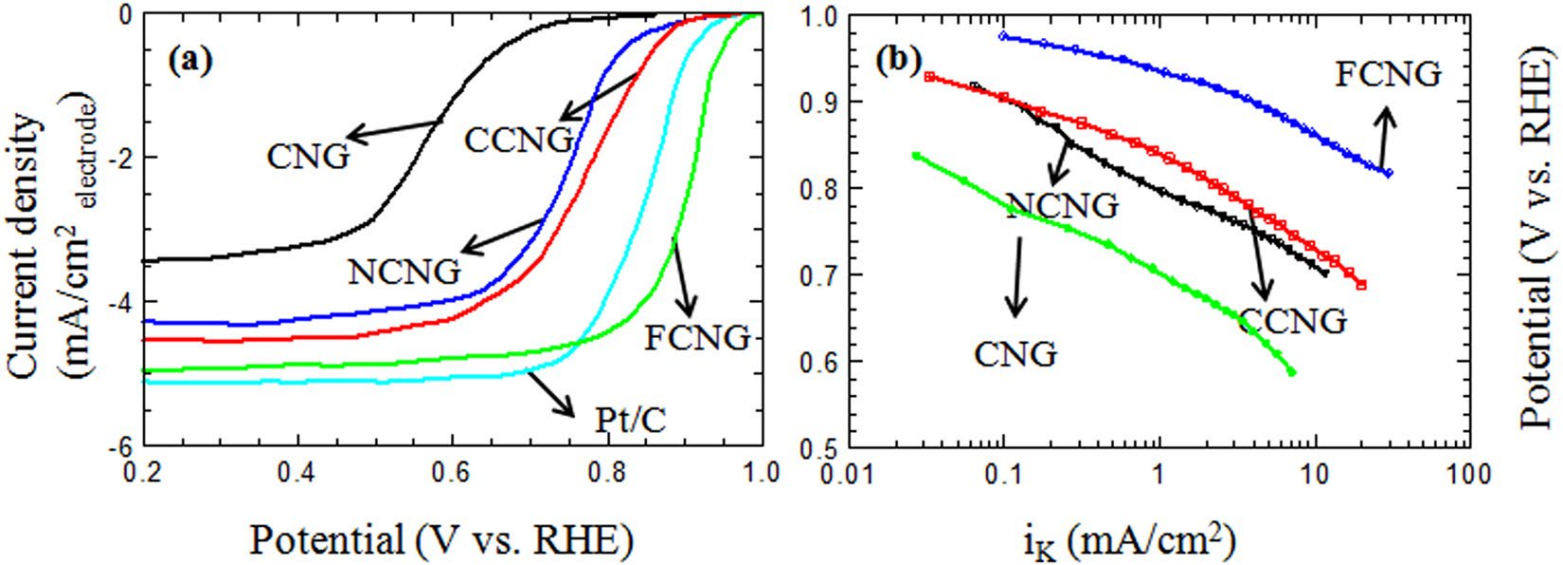
(**a**) Linear sweep voltammetry curves and (**b**) Tafel plots for ORR with a rotating speed of 900 rpm in O_2_ saturated 0.5 M H_2_SO_4_ solution, at potential scan rate of 5 mVs^−1^ and 25 °C on different carbon catalysts and the Pt/C reference catalyst using a RDE electrode.

The electrochemical analyses were performed on the metal free N-doped and the metal/N-doped carbon catalysts to evaluate the catalyst performance as the PEFC cathodes. Accordingly, linear sweep voltammetry curves (LSV) and the Tafel plots show the electrocatalytic activity of different carbon catalysts and also a Pt/C reference electrode (10 wt% E-TEK, 200 µg_pt_/cm^2^) toward the ORR in Fig. 6. Moreover, the related data on the LSV results are presented in Table 1. All the catalysts are catalytically active toward oxygen reduction as shown in the ORR polarization plots with the ORR onset which is as high as 0.86 V (versus the reversible hydrogen electrode, RHE) for the metal free N-doped CNG carbon catalyst. A more considerable improvement in the ORR activity is achieved by insertion of the transition metals of iron, cobalt and nickel within carbon structure as evidenced by the positive shifts in the onset ORR potentials of 0.94, 0.97 and 1.00 V for NCNG, CCNG and FCNG, respectively, in addition to the enhancements in the obtained current densities for the metal/N-doped carbon catalysts.

As observed in LSV curves (Fig. 6a), the steep decline in low current density region for all the catalysts and specifically for the FCNG sample is less compared to other NPMCs and even the Pt/C catalyst. Accordingly, the Tafel slopes are expected to show small values for all the carbon catalysts, as a representative of high ORR activities and extremely low kinetic overpotentials. Table 1 summarizes the onset potentials (obtained from Fig. 6a), the Tafel slopes and the exchange current densities (obtained from Tafel equation^21,38^, *η* = *b*log(*i*/*i*_0_)) of the ORR on different catalysts. The Tafel plots on different carbon catalysts are presented in Fig. 6b to compare their activity according to their kinetic current densities by correcting the mass transport. It is evident that the activity of catalysts increases in the order:

$${\rm{FCNG}} > {\rm{CCNG}} > {\rm{NCNG}} > {\rm{CNG}}$$

The Tafel slope values of all the carbon catalysts are almost similar to that of our Pt/C reference electrode with a high catalyst loading of 200 µg cm^−2^. These small values confirm the fast kinetic of the transfer of electrons on the catalyst surfaces. However, the current densities for the carbon catalysts are less than that of the Pt/C reference electrode. It is due to the large thickness of each carbon catalyst onto glassy carbon electrode (1 mg cm^−2^ against 0.2 mg cm^−2^ for Pt/C) which causes high mass transfer and electrical resistant, resulting in lowering the current density^11,39^.

**Figure 7:**
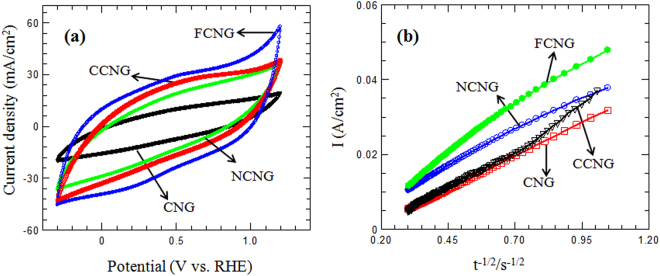
(**a**) Cyclic voltammograms of the N-doped and metal/N-doped carbon catalysts in Ar-saturated 0.5 M H_2_SO_4_ at 50 mV/s. (**b**) Plots of *I* vs. *t*^−1/2^ for oxygen reduction of different carbon catalysts.

The exchange current densities for all the catalysts are nearly the same, although for the iron and cobalt doped catalysts, the *i*_0_ values are slightly higher (Table 1). Nevertheless, the *i*_0_ values in comparison with other NPMCs are at least three orders of magnitude higher and they are comparable with that for our high loading Pt/C catalyst^9,38,40^. Figure 7a shows the cyclic voltammetry curves in Ar-saturated 0.5 M H_2_SO_4_ for the metal/N-doped and the metal free N-doped carbon catalysts. The voltammograms are virtually featureless, but with wide curvatures which are attributed to the characteristic changes of reduction/oxidation (redox) states of the surface groups of the catalyst and due to Faradic reaction occurring between the nitrogen atoms of the sample and H^+^ in the electrolyte^9,40–42^. The lack of sharp peaks in the carbon catalyst voltammograms implies the absence of the free metal particles onto the carbon catalyst surfaces^9^, although the CV of the FCNG catalyst reveals a pair of very broad redox peaks centered at *ca*. 0.5 V. This may be expected for either one-electron redox of the surface quinone-hydroquinone groups or the change in the oxidation state of iron species as Fe^3+^/Fe^2+^ redox on the catalyst surface according to the result obtained in the XPS analysis^9^.

Consistent with the modified Cottrell equation, chronoamperometry was used to determine the oxygen permeability in various electrodes^21^. Permeability was determined as the product of *D*_*b*_^*1/2*^*c*_*b*_, where *D*_*b*_ is the diffusion coefficient and *c*_*b*_ is the concentration of oxygen. As observed in Table 1 and Fig. 7b, the permeability for the FCNG catalyst is higher than that of other catalysts in agreement with the result obtained for the exchange current densities (*i*_0_). However, the oxygen permeability values show only a small difference among the metal free and metal/N-doped carbon catalysts. This reflects the porous nature of each catalyst and the abundant presence of active sites on the catalyst surfaces, resulting in a fast permeation and reduction of oxygen on the catalyst surfaces. In spite the large thickness of the carbon electrodes, they show slightly smaller values of permeability in comparison to that of the Pt/C electrode.

## Discussion

In our previous works, we used three types of organic-inorganic nanohybrids (with different organic anions) as the layered nanoreactors to produce NCM nanosheets. We showed that the NCMs with tunable nitrogen contents could be obtained by our method and they could be used as high performance electrocatalysts for the ORR and highly efficient material in supercapacitors^21,22^. In spite of the works done by other authors, the method does not need the use of the organic chemicals such as macrocycles, chelates, metal-organic frameworks, ionic liquids (ILs) and polymers, etc which contain nitrogen or metal/nitrogen elements for production of the NPMCs^2,4,11–13^. Specifically, each organic anion that can only be intercalated between ZHN layers is an appropriate candidate for production of the NCMs and metal/N-doped carbon catalysts. Why the use of a layered nanoreactor? As seen in this paper, practically all what is required for an excellent performance of the ORR in using NPMCs could be obtained in the carbon catalysts produced by this method. Accordingly, due to the morphology, these carbon catalysts with 3D nanoplate structure are capable of preventing the sheets from re-stacking and agglomerating when they are used in the electrodes as packed^43^. This 3D form provides high surface area and the relevant channels for easy transferring the oxygen gas on the surface of the carbon sheets which have pore sizes around 2 nm. These optimum sized pores are excellent sites to capture oxygen molecules for reduction and therefore obtaining higher ORR activity. They are not too small due to accessibility for oxygen molecules and also not too big thereby reducing the conductivity of the material^44–47^. As stated, doping each carbon plate with nitrogen and iron led to an increase in the surface area value without any change in the mean pore size and the pore size distribution. Unlike to our method, most researchers have used an impregnation method to add some transition metals within carbon structure by using the metal salt solutions followed by an extra heat-treatment process^9,25,33^. In addition to the increase in the number of the catalyst synthesis steps and therefore higher cost, this impregnation approach would result in a decrease in surface area value, porosity and total pore volume as a result of filling the pores by the metal salts^41^. Due to the nitrogen bond type in the carbon structure, it is really still under debate whether nitrogen type has the most important role in the ORR performance. What is known is that in general, those nitrogen atoms are positioned at the edge of the graphene sheets are more effective for the ORR than those positioned at the bulk^48,49^. It is well known that the pyridinic and pyrrolic nitrogen bonds are both positioned at the edge in spite of quaternary nitrogen that can be bonded with carbon at both bulk and edge positions in a graphene sheet^9,45,50^. Moreover, pyridinic nitrogen can donates only one electron to the aromatic system of the carbon ring due to its lone electron pair in sp^2^ orbital perpendicular to the p orbitals in the aromatic ring and one electron p_z_ orbital parallel to p orbitals in the ring. Some authors believe that this lone electron pair takes part in the ORR as the active sites^33,51^. In the pyrrolic nitrogen, the lone electron pair is in p_z_ orbital parallel to p orbital in the aromatic carbon ring and therefore can denote two electrons to the graphene π-system. Consequently, the pyrrolic nitrogen can increase the charge mobility of the graphitic lattice more than other nitrogen bond types^45^. Nevertheless, it was reported that, except for the case of N-X, all of the other nitrogen bond types play an important role in the ORR performance^52^. According to XPS results shown in Fig. 5, our method for preparation of the FCNG catalyst could result in the production of the highest percentage of pyrrolic and pyridinic nitrogen among other carbon-nitrogen bonds. High exposure of the gallate anions to nitrate anions between the layers of ZHN is expected to rendering more pyrrolic and pyridinic nitrogen, resulting in an enhancement of the ORR activity^12,23^. Moreover, the FCNG catalyst has a small amount of N-Q configuration. It has been reported that although N-Q does not help to ORR activity, it also does not suppress ORR activity^33^. It is while; the N-Q configuration improves the conductivity of carbon materials^34,53^. In addition, XPS analysis reveals the presence of oxygen element in a large percentage (≈9%), and also iron on the FCNG surface as Fe(II) and Fe(III) ions, but not in metallic form^12^. As the nitrogen contribution in Fe-N bond (398.82 eV) is about 0.68% (Table S2), therefore approximately half of iron content is in Fe–N_4_ form and the remaining half of the iron may be in oxide form due to acid washing the catalyst. It should be noted that the presence of the oxygen in accompanied with nitrogen in the carbon catalysts has a synergic effect toward the ORR as the carbon sheets with both O- and N-functionalities have shown 4e reduction pathway against a 2e reduction pathway for the sheets with only N-functionality. Moreover, it was reported that an improved ORR performance can be obtained for the graphene sheets with higher oxygen content. The presence of carbon-nitrogen, carbon-carbon and carbon-oxygen bonds in the FCNG catalyst was also confirmed via FTIR spectroscopy (Fig. S5b). As stated, these bonds are key factors to improve the electrocatalytic activity and facilitate the ORR performance^1,14,44,47^. Accordingly, if we accept that the nitrogen and iron dopants act as the active sites toward the ORR, one can deduce that an extremely uniform distribution of the dopants (as observed in Fig. 4) would result in an extra enhancement in the ORR activity, ensuing in the absence of the large kinetic overpotential for the catalyst. The disordered graphite structure of the FCNG catalyst may enhance the active site numbers via facilitating the incorporation of nitrogen and metals within carbon structure as observed by XPS analysis^9^. Moreover, the graphite structure can also improve the conductivity of the catalyst^1,54^. The elemental composition data obtained by EDS and CHNS analyses (Table 1) reveals that the nitrogen and metal percentages are almost the same among all the catalysts and therefore the electrochemical performance for each catalyst is due to the metal dopant type.

## Conclusion

In conclusion, this paper focuses on a new method to synthesize metal/N-doped carbon catalysts by using a layered nanoreactor. In this procedure, intercalation of gallate anions into the interlayer of the undoped and metal-doped ZHNs and then heat-treating of the obtained organic-inorganic nanohybrids at 800 °C under argon atmosphere could produce 3D metal/N-doped carbon nanosheets which were used as catalysts for the ORR. Simultaneously, high nitrogen content, highly porous structure with appropriate pore size and finally high ORR performance could be provided from an entirely new class of the organic-inorganic layered nanoreactors. The type of the organic compound used, organic/nitrate anions ratio between the ZHN layers, the used element dopant type and also the heat-treatment temperature are key factors that have impacts on the morphologies, carbon plate thickness, dopant content and porosity type of the obtained carbon materials. These parameters could change the ORR performance and they need to be taken into account in upcoming research works, improving our knowledge and insight into the factors contributing to the mechanisms and specifically the kinetics of the ORR for this class of materials. Moreover, some other *α*-phase metal hydroxides such as copper, magnesium, nickel, cobalt etc. can be used as the layered nanoreactors to produce carbon catalysts with various morphologies and different properties. Finally, this approach can simultaneously produce metal oxide/N-doped carbon nanohybrids (for instance, ZnO/N-doped carbon at 700 °C in this work) for use as the active materials in the PEFC cathodes, supercapacitors, batteries and gas storage materials.

## Methods

### Catalyst synthesis

The initial zinc hydroxide nitrates (ZHNs) were synthesized from 0.2 M Zn(NO_3_)_2_ solution without or with 2% (molar in mother liquor) Fe^2+^ (FZHN), Co^2+^ (CZHN) and Ni^2+^ (NZHN)^1^. The solutions were kept at pH 7.0 ± 0.05 by dropwise additions of 0.5 M NaOH solution with vigorous stirring. The precipitates were filtered, washed with water and acetone and dried in an oven, overnight at 80 °C. Zinc hydroxide nitrate/gallate nanohybrids (incompletely ion-exchanged organic anion) were prepared using ion-exchange method by contacting 1 g of the as-synthesized ZHN (or metal-doped ZHN) into 250 mL solution of 0.05 M gallate for a half-hour under nitrogen atmosphere, and, labeled as ZHNG, FZHNG, CZHNG and NZHNG for the metal dopant free and iron, cobalt and nickel doped organic-inorganic nanohybrids, respectively^27^. The zinc hydroxide gallate nanohybrids (completely ion-exchanged organic anion) were prepared by contacting 1 g of the ZHN (or metal-doped ZHN) into 250 mL solution of 0.2 M gallate for two hours under nitrogen atmosphere, and, labeled as ZHG, FZHG, CZHG and NZHG for the metal dopant free and iron, cobalt and nickel doped nanohybrids, respectively. In this study, the formation of gallate anion was done by dissolving gallic acid in distilled water, bringing the resulting solution to pH ≈ 6 by adding 2 M NaOH^1^. The nanohybrids obtained after filtering, washing and drying were heated at 800 °C in an electric tubular furnace under argon atmosphere at a flow rate of 50 ml/min for 1 h at a rate of 5 °C/min and then cooled down naturally under argon atmosphere. To obtain the carbon catalysts and the remove any excess metals (or metal oxides), the heat-treated products were leached in 1 M HCl (1 g solid/200 ml) at 50 °C for 3 hours, thoroughly washed in distilled de-ionized water and acetone and finally dried at 80 °C, overnight^21,27^. The dopant free carbon material and the metal free N-doped carbon catalyst derived from, respectively, zinc hydroxide gallate and zinc hydroxide nitrate/gallate nanohybrids were denoted as CG and CNG, where the metal/N-doped carbon catalysts derived from the metal-doped zinc hydroxide nitrate/gallate nanohybrids were labeled as FCNG for iron/N, CCNG for cobalt/N and NCNG for nickel/N-doped carbon catalysts.

### Physical characterization

X-ray diffraction patterns were collected on a Philips PW1730 powder diffractometer unit using CuK_α_ (*λ* = 1.54 Å) at 10 kV and 10 mA. FTIR spectra were recorded using a Perkin-Elmer RXI spectrophotometer in the range of 400–2000 cm^−1^. CHNS instrument (Leco, USA) was used to determine the mass percentages of nitrogen and carbon in the catalysts. Catalyst morphologies and the elemental composition and mapping were characterized by the field emission scanning electron microscope and energy-dispersive X-ray spectroscopy (ZIESS Supra 55VP and JEOL JFM-6700F). Thermal properties were determined using a Mettler Toledo TGA/SBTA851e and a simultaneous thermal analyzer instrument (STA), Polymer Laboratories PL-STA1640, conducted under argon atmosphere at a flow rate of 50 ml/min from 25 to 850 °C at a rate of 5 °C/min. The surface area and pore size analyses were determined using BELSORP measuring instruments (BELSORP-mini, JAPAN, INC.) using nitrogen gas adsorption-desorption technique at 77 K. X-ray photoelectron spectroscopic (XPS) measurement was performed on a Thermo Scientific K-Alpha X-ray photoelectron spectroscope using Al K_*α*_ and spot size 400 *µ*m.

### Electrochemical tests

Electrochemical tests were carried out using an EG&G potentiostat (PARSTAT 2273). The ORR activity of the carbon catalysts was evaluated using a rotating ring-disk electrode (RDE) setup with the standard three compartment electrochemical cell, a glassy carbon disk (4 mm in diameter) as a working electrode, a graphite rod as the counter-electrode (to avoid any contamination of platinum counter electrode)^9^ and a saturated calomel electrode (SCE) as a reference electrode was inserted close to the working electrode to preserve the working electrode’s potential at a fixed value. All potentials were then referenced to a normal hydrogen electrode (NHE). The catalyst inks were prepared by ultrasonically dispersion (45 min) of the carbon catalysts in an alcoholic solution containing Nafion solution (5 wt% solution) and ethanol (2 mL)^55^. The ink was then dropped onto the glassy carbon to obtain a catalyst loading of 1 mg cm^−2^ and dried in the air. The LSV polarization plots were recorded in an O_2_-saturated 0.5 M H_2_SO_4_ electrolyte in potential range of −0.3 to 1.2 V (NHE) at scan rate of 5 mV/s. Cyclic voltammetry measurements were carried out in an Ar-saturated 0.5 M H_2_SO_4_ electrolyte in potential range of −0.3 to 1.2 V (NHE) at a scan rate of 50 mV/s and were recorded after 15 scans for each sample. Chronoamperograms were obtained by holding the potential of the electrodes at 0.3 V for 10 s after holding the potential of the electrodes at 1.2 V for 60 s^21^.

## Electronic supplementary material

Supplementary Information
