# Supplementary material for: Three dimensional metal/N-doped nanoplate carbon catalysts for oxygen reduction, the reason for using a layered nanoreactor
Source: Sci Rep. 2018 Feb 21;8:3404. doi: 10.1038/s41598-018-21782-3 (PMC5821842; doi:10.1038/s41598-018-21782-3)
Supplement: Supplementary file 1 — Supplementary Information [file 41598_2018_21782_MOESM1_ESM.pdf]

<u>**Supplementary information**</u>

Three dimensional metal/N-doped nanoplate carbon catalysts for oxygen reduction, the reason for using a layered nanoreactor

Mohammad Yeganeh Ghotbi[1]*, Arash Javanmard[1] and Hassan Soleimani[2]
[1]Materials Engineering Department, Faculty of Engineering, Malayer University, Malayer, Iran
[2]Fundamental and Applied Science Department, Universiti Teknologi PETRONAS, 31750 Seri Iskandar, Malaysia

---

* Corresponding author: Tel.: + 98-8133339841.

E-mail address: m.yeganeh@malayeru.ac.ir, yeganehghotbi@gmail.com

Thermal and phase stability behavior of the FZHN precursor and its FZHNG nanohybrid

Figure S2 shows TG/DTG curves for the FZHN, organic compound (gallic acid), the FZHG gallate nanohybrid and the FZHNG nitrate/gallate nanohybrid. As shown in the figure, FZHN decomposes in two major steps and a minor step. The initial major step is owed to the removal of the intercalated water molecules with respective weight loss of 5.5% at temperature maximum of 88 ˚C as following,

$Zn_5 (OH)_8 (NO_3)_2.2H_2O \rightarrow Zn_5 (OH)_8 (NO_3)_2 + 2 H_2O$ (1)

with theoretical weight loss of 5.8 %. The minor step (it can be seen as a shoulder in the DTG curve) at about 150 ˚C is attributed to the converting of anhydrous ZHN to another type of ZHN ($Zn_3(OH)_4(NO_3)_2$, card No: 52-0627 with 0.69 nm basal spacing)[1] and a small amount of zinc oxide with theoretical weight loss of 6.1 % as following,

$Zn_5 (OH)_8 (NO_3)_2 \rightarrow Zn_3 (OH)_4 (NO_3)_2 + 2 ZnO$ (2)

For our sample, this weight loss is 7.0 %. The second major step is due to the collapsing the layered material and production of zinc oxide with respective weight loss of 28 % at temperature maximum of 173 ˚C. The equation for this step is as following,

$Zn_3 (OH)_4 (NO_3)_2 \rightarrow 3 ZnO + 2 NO_2 + ½ O_2 + 2 H_2O$ (3)

with theoretical weight loss of 25.6 %.

Therefore, the sum of the theoretical weight losses is 37.5 % and for our sample is 40 %. It shows a good agreement between theatrical and practical data for thermal behavior of the FZHN. Meanwhile, it should be noted that some difference between theoretical and practical values is due to the removal of the surface adsorbed water molecules for the FZHN. Overall, this TG curve in consistent with XRD data confirm that the layered hydroxide cannot keep its structure stable at the temperatures higher than 200 ˚C. However, by FTIR analysis, it was observed that

the nitrate anions can still be intercalated between zinc oxide layers mostly in the stable state at temperature as high as 400 ˚C and only at the temperatures higher than 400 ˚C, nitrate anions are decomposed and released as NO_2 gas molecules[2]. This is evidenced by observation of the continued weight losses in the FZHN sample at temperatures higher than 200 ˚C to 800 ˚C.

Gallic acid decomposes by heat-treatment under inert gas in two steps with the weight losses of 44.0 and 25.0 % at temperatures maxima of 264 and 319 ˚C, respectively (Fig. S2b). The release of CO_2 by decomposition of acidic group of gallic acid, and, also the decomposition of phenolic groups along with production of water is assumed to be the reasons to observe the first weight loss step of gallic acid[2,3] with theoretical weight loss of 44.1 % according to following equation in good agreement to our obtained result.

$$2C_7H_6O_5 \rightarrow C_{12}H_6O_3 + 2CO_2 + 3H_2O \qquad (4)$$

The second weight loss can be due to decomposition of benzene ring to carbon with theoretical weight loss of 23.5 %.

$$C_{12}H_6O_3 \rightarrow 4C + 3CO + C_5H_6 \qquad (5)$$

As observed, gallic acid cannot be stable at temperatures higher than 320 ˚C and the obtained residue carbon is 13 % at 800 ˚C (2C is remained from 7C due to gallic acid). Figure S2c shows the TG/DTG curves for the FZHG. Three major steps of the weight losses of 5.5, 27.0 and 27 % occurring at temperatures maxima of 80, 590 and 795 ˚C are observed. Except the first weight loss due to the removal of the surface adsorbed water molecules at about 80 ˚C, any major weight loss cannot be seen up to around 600 ˚C in the TG curve. Given that the surface adsorbed water weight loss is 5.5 % and the intercalated water weight loss is theoretically considered to be 4.2 % (a total of ≈10 %), 10 % weight loss is equal to 300 ˚C in the TG curve and, therefore, the next step weight loss occurred from around 300 ˚C. In accordance with XRD patterns for the

heat-treated products of the FZHG (Fig. S3a), the weight loss at 300 ˚C and at higher temperatures is due to the dehydroxylation process. In fact, the dehydroxylation started gradually around 300 ˚C and completed at around 600 ˚C. As shown in Fig. S3a, XRD pattern confirms that the layered structure can still be observed for the FZHG heat-treated at 600 ˚C. Moreover, FTIR spectra for the heat-treated products of the FZHG (Fig. S4a) show that gallate anion peaks are similar to those of the unheated nanohybrid, but with less intensities and some red shifts. This confirms that gallate anions positioned between the inorganic layers are not completely decomposed even at 600 ˚C. However, the dehydroxylation weight loss ($Zn_5(OH)_8(C_7H_5O_5)_2$ to $Zn_5O_5(C_7H_5O_5)_2$) should be 10.6 % (theoretically) and the obtained weight loss at 600 ˚C is 27.0 %. It means that the second weight loss for the FZHG is due to both dehydroxylation and a follow up of the first step decomposition of gallic acid. Accordingly, the presence of gallate anion between zinc hydroxide layers could profoundly enhance thermal resistance not only for organic compound, but also for the layered hydroxide structure. Complete decomposition of gallate anions and producing carbon material is obtained at the temperatures higher than 600 ˚C (see Figs. S3a and S4a). Alternatively, a further major weight loss is considered at around 790 ˚C in the TG/DTG curve of the FZHG. If we suppose that the heat-treatment of the gallate anions inside the FZHG layers would produce carbon material in the same manner as that of free gallic acid according to following formula,

$$Zn_5(OH)_8(C_7H_5O_5)_2 \rightarrow 5\ ZnO + 4C + \text{volatile components} \qquad (6)$$

all the residual material should be 57 %. It is while; we can see that the residual product is about 40 % for the FZHG after heat-treatment at 850 ˚C. According to TG curve (Fig. S2c) and XRD data for the FZHG (Fig. S3b), one can deduce that a new event happens at temperatures higher than 700 ˚C. This is the reduction of ZnO to metallic zinc and its evaporation on the surface of

the produced carbon[4-6]. Nevertheless, the evaporation does not occur for the dopant metals of Ni, Co and Fe and, they could be only reduced to metallic form as evidenced by the XRD patterns of the metal-doped nanohybrids heat-treated at 800 ˚C (Fig. S3b)[6]. According to data shown in Fig. S3b, it is worthwhile to mention that the presence of the metal dopant agents could catalyze the reduction and evaporation of ZnO. Accordingly, ZnO peaks are completely being eliminated in the XRD patterns and FTIR spectra (Fig. S4a) for the heat-treated products of metal doped layered nanohybrids of NZHG, CZHG and FZHG at 800 ˚C, but not for the undoped ZHG nanohybrid. Additionally, the EDS analysis was performed on the CZHG and FZHG samples heat-treated at 800 ˚C to confirm the elemental compositions of the produced metals as observed in their XRD patterns. The skewer-like metallic cobalt (obtained via fusion of the melted cobalt nanoparticles) and carbon nano plates are seen in the FESEM image of the heat-treated CZHG sample (Fig. S5a). Also, EDS data shows some dissolved oxygen, carbon and metallic zinc for the obtained metallic cobalt and iron (Figs. S5b-f).

For the iron-doped zinc hydroxide nitrate/gallate nanohybrid (FZHNG), we used a STA to get better insight into the reaction between nitrate and gallate anions inside the inorganic layers. As shown in Fig. S2d, there are two minor weight losses of 2.9 and 8.6 % at temperatures maxima of 96.6 and 167.7 ˚C. The first one is due to the removal of the adsorbed water and the second one is owed to the dehydroxylation process for the zinc hydroxide nitrate moiety in each nanohybrid plate (see Fig. S6a and Fig. S1). This small amount of the dehydroxylation weight loss (8.6 %) demonstrates that only dehydroxylation process occurs at this temperature, but not the decomposition of nitrate anions along with the release of NO_2. It means that the nitrate anions are present mostly between zinc oxide layers encapsulated by the zinc hydroxide gallate layers. DTA analysis confirms the two mentioned endothermic processes. At around 400 ˚C,

there is an exothermic process followed by an intense endothermic process accompanied to the main weight loss of 32 % occurring at temperature maximum of 432 ˚C. The exothermic process can be due to the reaction between nitrate and gallate anions, resulting in the burning some organic anions and also starting the formation of nitrogen doped carbon material. In fact, during the heat-treatment process, the basal spacing of the nanohybrids acts as a layered nanoreactor for the reaction between gallate and nitrate moieties to produce N-doped carbon catalysts[7,8]. The inorganic layers can shield the content of the basal spacing (gallate and nitrate) from direct heat and thus prevent the evaporation and burning out the anions. As seen in Fig. S2d, unlike to free gallic acid and FZHG nanohybrid, gallate anions inside the FZHNG layers are not decomposed in two steps, but in one step due to the gallate-nitrate reaction. An endothermic reaction can also be observed after 700 ˚C due to the reaction between the produced carbon and zinc oxide materials as following,[5]

$$ZnO_{(s)} + C_{(s)} \rightarrow Zn_{(g)} + CO_{(g)} \qquad (7)$$

Due to phase stability of the materials during the heat-treatment process and confirming those events observed in the thermal analysis for the FZHN and its FZHNG nanohybrid, an in-situ heating XRD instrument was used to monitor the structural degradation of the samples in the temperature range from 25 to 800 ˚C at a constant heating rate of 5 ˚C min^{-1} and an Ar flow rate of 50 mL min^{-1}. Figure S6a shows that the FZHN heated at 150 ˚C is composed of three-phase materials, original FZHN with slightly smaller basal spacing due to the removal of the intercalated water molecules, another type of zinc hydroxide nitrate ($Zn_3(OH)_4(NO_3)_2$), and also some collapsed ZHN material (zinc oxide) in agreement with literature and TG analysis[1]. The heat-treated products in the range of 300 to 800 ˚C include pure phase zinc oxides (Card 70-2551), as their XRD peak intensities are increasing with the increase in the calcination

temperature due to the growth of the particles. For the FZHNG sample at 25 ˚C, the XRD pattern shows a bi-basal spacing layered hydroxide material including the nitrate anion (0.97 nm) layers inside the gallate anion (0.88 nm) layers. The material heated at 150 ˚C shows that zinc hydroxide nitrate layers are completely collapsed (dehydroxylation) due to the lack of ZHN basal spacing peak and the presence of the zinc oxide peaks. It is while; zinc hydroxide gallate layers are present unchanged in consistent with the TG analysis at this temperature. At higher temperature of 300 ˚C and even at 600 ˚C, zinc hydroxide gallate basal spacing peak can still be observed, although most gallate anions are decomposed at temperatures lower than 600 ˚C due to the obtained weight loss at this temperature (see Fig. S2d). A new peak can also be seen for the heat-treated product at 800 ˚C as well as zinc oxide peaks. This new peak is attributed to the metallic iron produced by carbothermal reduction of the metallic dopant, iron oxide. The intensity of the zinc oxide peaks are diminished at 800 ˚C due to the reduction and evaporation of some ZnO particles that have access to the carbon material, similar to the FZHG sample heated at the temperature (Fig. S3b).

References

S1. Hussein, M. Z. b., Yeganeh Ghotbi, M., Yahaya, A. H. & Abd Rahman, M. Z. The effect of polymers onto the size of zinc layered hydroxide salt and its calcined product. *Solid State Sci.* **11**, 368-375 (2009).

S2. Yeganeh Ghotbi, M., bin Hussein, M. Z., Yahaya, A. H. & Abd Rahman, M. Z., Thermal decomposition pathway of undoped and doped zinc layered gallate nanohybrid with Fe^{3+}, Co^{2+} and Ni^{2+} to produce mesoporous and high pore volume carbon material. *Solid State Sci.* **11** 2125-2132 (2009).

S3. Xu, Z. P. & Zeng, H. C. Decomposition processes of organic-anion-pillared clays $Co_aMg_bAl(OH)_c(TA)_d{\cdot}nH_2O$. *J. Phys. Chem. B* **104**, 10206-10214 (2000).

S4. Yang, P. *et al.* Controlled growth of ZnO nanowires and their optical properties. *Adv. Func. Mater.* **12**, 323-331 (2002).

S5. Tzouganatos, N., Dellamico, M., Wieckert, C., Hinkley, J. & Steinfeld, A. On the development of a zinc vapor condensation process for the solar carbothermal reduction of zinc oxide. *Min. Met. Mater. Soc.* **67**, 1096-1109 (2015).

S6. Wang, X. *et al.* Directly converting Fe-doped metal-organic frameworks into highly active and stable Fe-N-C catalysts for oxygen reduction in acid. *Nano Energy* **25**, 110-119 (2016).

S7. Yeganeh Ghotbi, M., Feli, B., Azadfalah, M. & Javaheri, M. Ultra high performance N-doped carbon catalysts for the ORR derived from the reaction between organic-nitrate anions inside a layered nanoreactor. *RSC Adv.* **5**, 92577-92584 (2015).

S8. Yeganeh Ghotbi, M. & Azadfalah, M. Design of a layered nanoreactor to produce nitrogen doped carbon nanosheets as highly efficient material for supercapacitors. *Mater. Design* **89**, 708-714 (2016).

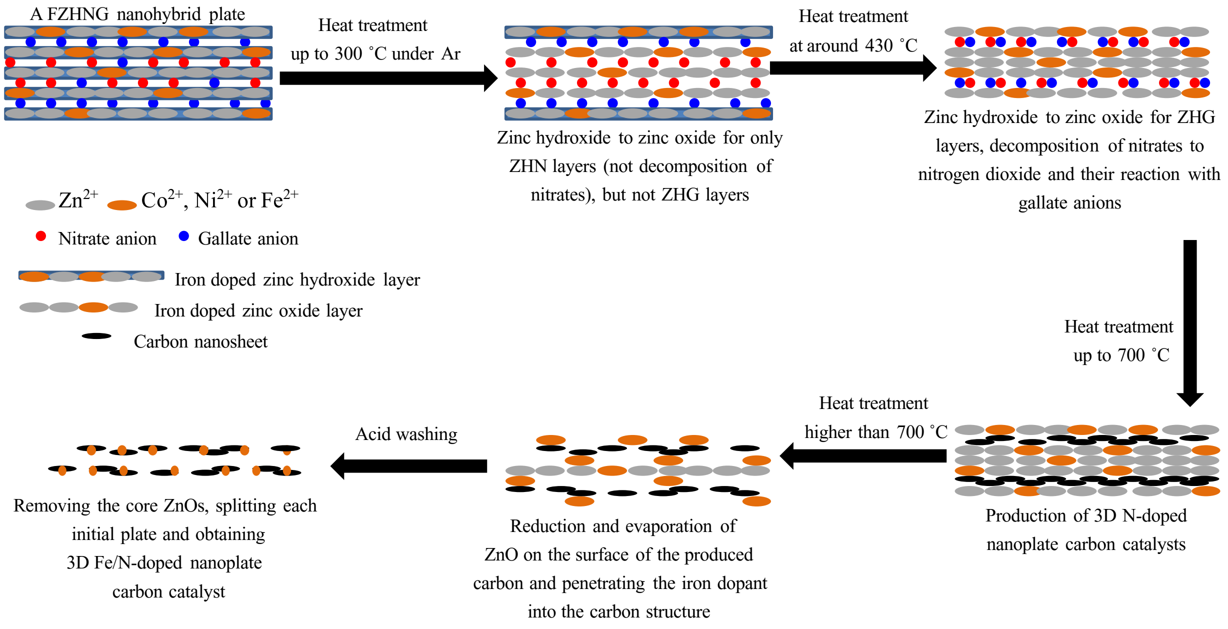

Figure S1 | Schematic diagram of the heat-treatment process of a FZHNG nanohybrid plate.

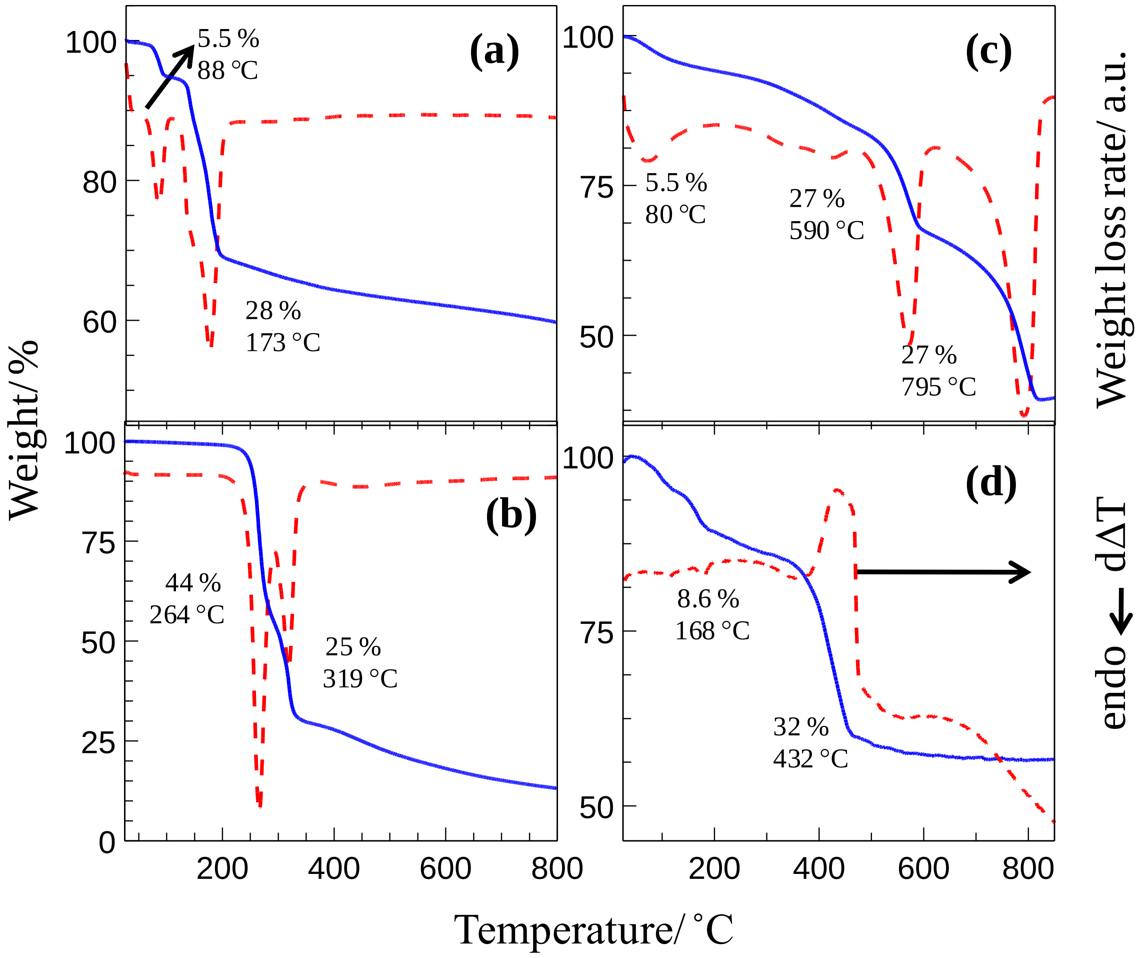

Figure S2 | TG/DTG curves for the FZHN (a), gallic acid (b), FZHG nanohybrid (c) and TG/DTA curves for FZHNG nanohybrid (d).

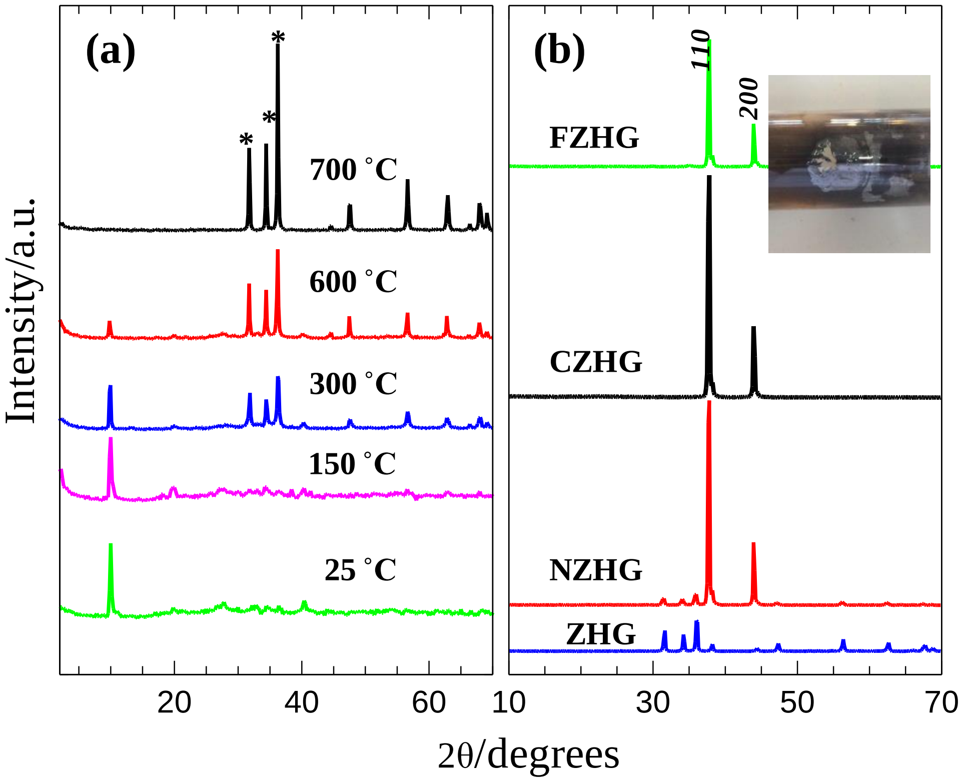

Figure S3 | XRD patterns for the heat-treated products of FZHG nanohybrid at various temperatures (a) and the heat-treated products of ZHG, NZHG, CZHG and FZHG nanohybrids at 800 ˚C (‘*’ indicates ZnO phase). Inset in (b) shows a photograph of the metallic zinc deposited on the inner surface of a colder zone of the tube furnace.

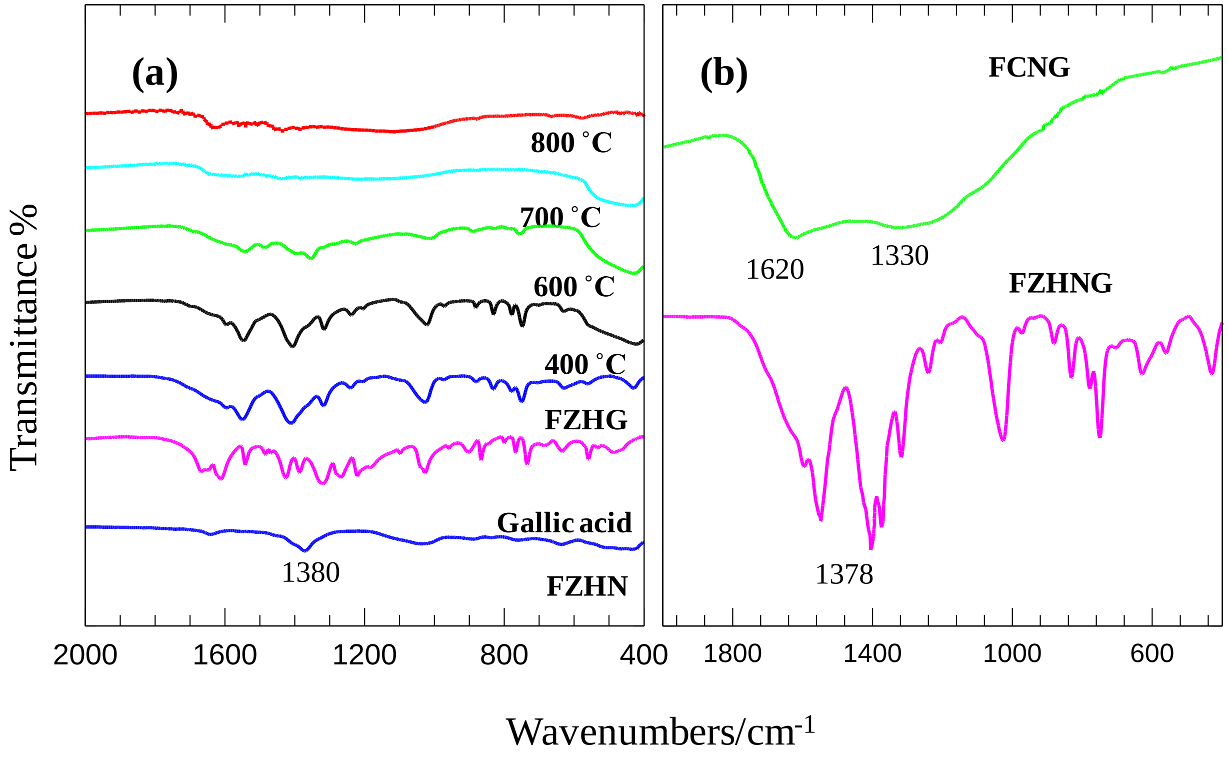

Figure S4 | FTIR spectra for the FZHN precursor, gallic acid, the FZHG nanohybrid, the heat-treated products of FZHG at various temperatures (a) and the FZHNG nanohybrid and the FCNG carbon catalyst (b).

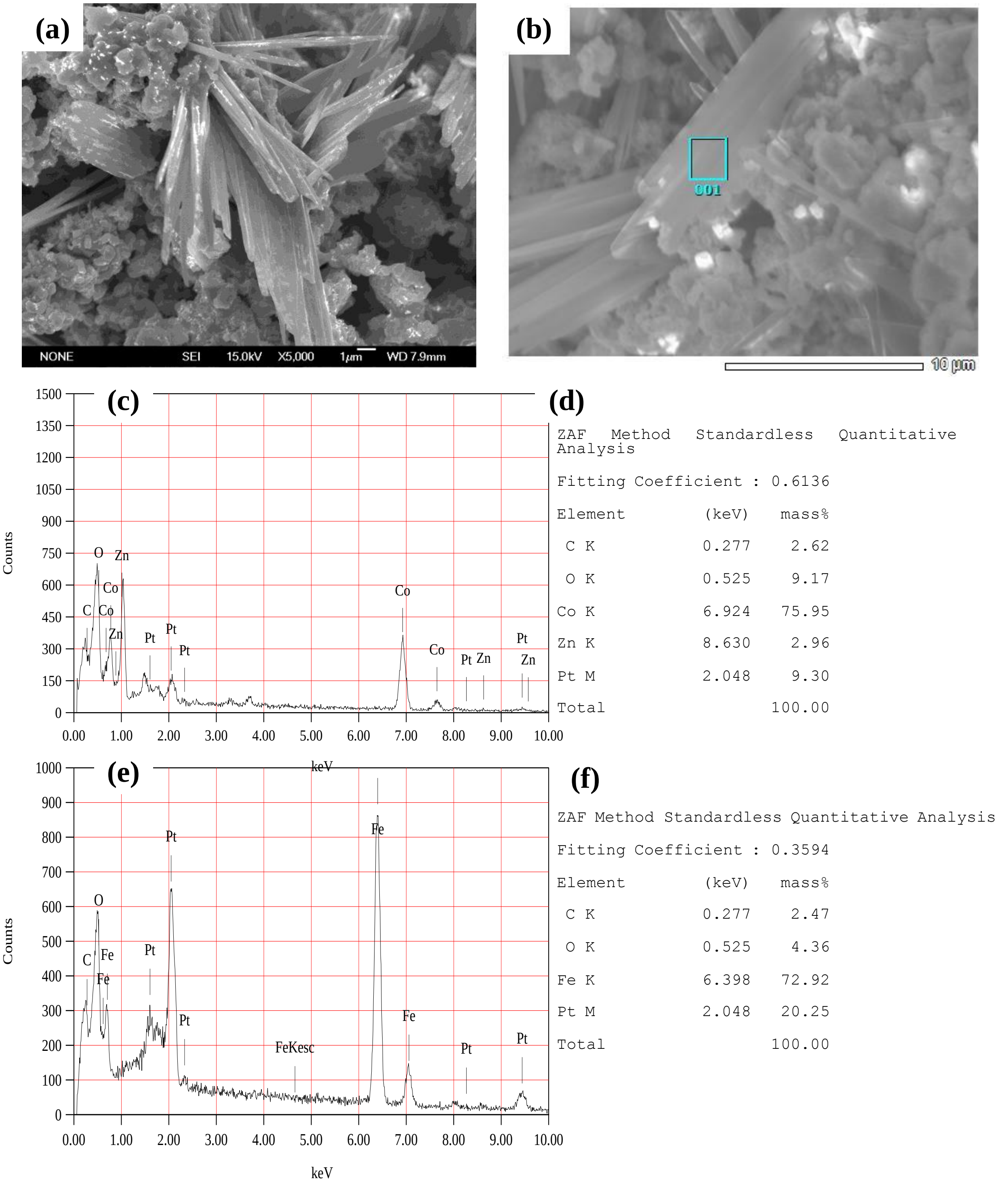

Figure S5 | FESEM image of the CZHG sample heat-treated at 800 ˚C (a) and EDS analysis data for the CZHG (b-d) and the FZHG (e and f) samples heat-treated at 800 ˚C.

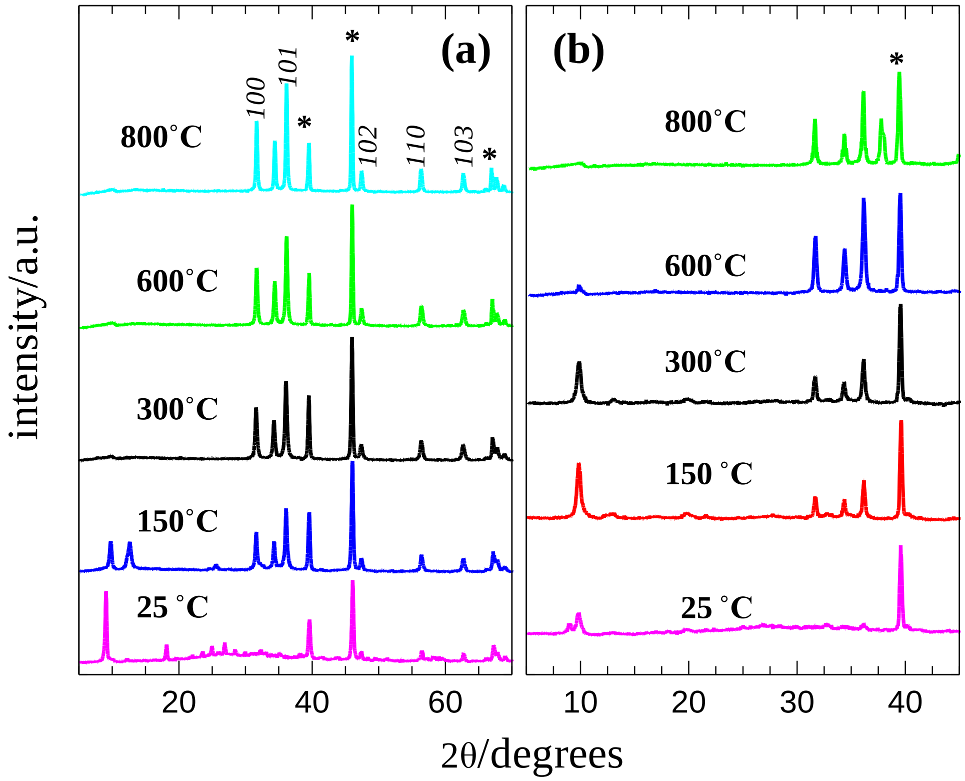

Figure S6 | XRD patterns for the heat-treated products of FZHN (a) and its FZHNG nanohybrid at various temperatures (b) ('*' indicates Pt sample holder peaks).

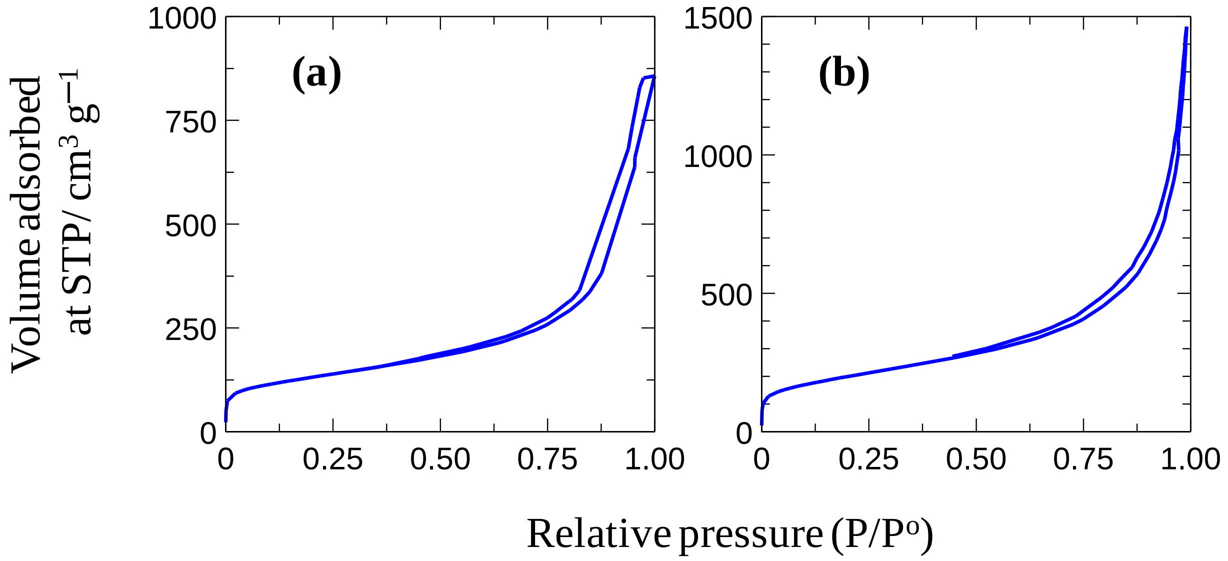

Figure S7 | Adsorption-desorption isotherms of nitrogen gas for the CG (a) and the FCNG (b) materials.

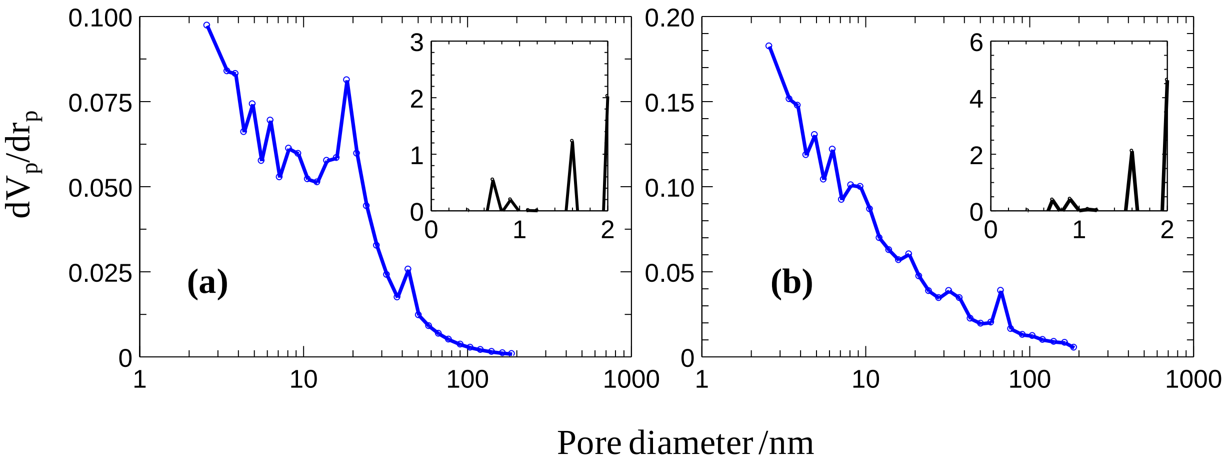

Figure S8 | BJH desorption pore size distributions for the CG (a) and the FCNG (b) materials (insets are MP-plots).

Table S1 | Obtained data from surface analyses of the CG and FCNG samples.

Sample	Surface area [$m^2.g^{-1}$]	Total pore volume [a], V_t [$cm^3.\ g^{-1}$]	Average pore diameter [nm]	$V_{meso + macro}$ [b] [$cm^3.\ g^{-1}$]	V_{micro} [$cm^3.\ g^{-1}$]
CG	458 [a]<br>426[e], 472[f]	1.26	11.0 [a],<br>2.6 [d], 2.0 [e]	1.24	0.02 [c]<br>0.0 [e], 0.01 [f]
FCNG	710 [a], 729 [e],<br>697 [f]	2.23	12.6 [a],<br>2.6 [d], 2.0 [e]	2.22	0.001 [c]<br>0.0 [e], 0.0 [f]

[a] Calculated by BET method, [b] by BJH method, [c] $V_{micro} = V_t - V_{meso + macro}$, [d] $d_{p,peak\ (BJH)}$, [e] by MP-Plot method and [f] by t-plot method.

Table S2 | Peak deconvolution analysis of N 1s and Fe 2p and their composition ratios for the FCNG catalyst.

Nitrogen	Peak BE	At. %	Iron	Peak BE	At. %
N1s Scan A	398.82	10.0	Fe2p Scan A	711.16	27.0
N1s Scan B	400.18	58.7	Fe2p Scan B	725.36	29.6
N1s Scan C	398.12	15.7	Fe2p Scan C	713.85	24.6
N1s Scan D	401.43	5.9	Fe2p Scan D	719.72	18.8
N1s Scan E	403.4	9.7			
